# Early-Stage Co-Creation and Human-Centered Development of Digital Therapeutics for Cognitive Rehabilitation in Early Neurocognitive Disorders: Convergent Mixed Methods Study

**DOI:** 10.2196/98625

**Published:** 2026-11-03

**Authors:** Federica Rossetto, Francesca Borgnis, Chiara Mangione, Claudia Serenelli, Sara Isernia, Francesca Baglio

**Affiliations:** 1 IRCCS Fondazione Don Carlo Gnocchi, Via Capecelatro 66, Milan, 20148, Italy, +39 0240308952

**Keywords:** mild cognitive impairment, subjective cognitive decline, digital therapeutics, human-centered design, mixed methods research, co-creation, cognitive rehabilitation, telerehabilitation

## Abstract

**Background:**

Subjective cognitive decline (SCD) and mild cognitive impairment (MCI) represent critical windows for early neurocognitive intervention, although access to timely, personalized rehabilitation remains limited. Digital health technologies offer opportunities to deliver scalable and individualized cognitive care; however, their successful implementation depends on an understanding of users’ needs, expectations, and barriers. Within a human-centered design (HCD) framework, actively involving end users during the earliest stages of development is essential to ensure that digital rehabilitation is acceptable, usable, and responsive to real-world rehabilitation needs.

**Objective:**

This study explores the experiences, rehabilitation needs, perceptions, and challenges of individuals with SCD and MCI regarding cognitive telerehabilitation. The findings are intended to inform the early-stage co-creation and human-centered development of digital rehabilitation solutions.

**Methods:**

The study is framed within an HCD perspective to inform the user-centered development of digital rehabilitation solutions. A convergent mixed methods design was adopted to integrate quantitative assessments with qualitative interviews. The semistructured interview explored 4 thematic domains: neurocognitive abilities, network support, user experience and engagement, and digital divide. Twenty-three individuals with SCD or MCI completed the interview together with measures including the Montreal Cognitive Assessment (MoCA), Cognitive Failures Questionnaire (CFQ), and an ad hoc Technological Expertise questionnaire. Qualitative interview data were analyzed using directed content analysis based on 4 predefined thematic domains by 2 independent raters, with interrater agreement assessed using Cohen κ. Qualitative and quantitative findings were subsequently integrated through exploratory Mann-Whitney *U* tests.

**Results:**

Twenty-three participants (n=16 female participants; mean age 74.3, SD 7.35 y) were included. Participants reported preserved cognitive performance (MoCA: mean 24.9, SD 3.23), low-to-moderate perceived cognitive difficulties (CFQ: mean 22.4, SD 9.48), and moderate technological familiarity. Qualitative findings revealed memory (n=12, 52.2%), attention/executive (n=6, 26.1%), and language (n=4, 17.4%) difficulties during everyday activities, together with widespread use of compensatory strategies. Participants also described emotional vulnerability, social withdrawal, and feelings of misunderstanding, as well as delayed rehabilitation access despite reporting cognitive concerns to health care professionals. Although most participants had limited prior knowledge of telerehabilitation, they expressed a positive attitude toward home-based rehabilitation, valuing its convenience, flexibility, autonomy, and accessibility when appropriate technological and clinical support was available. Concerns mainly related to limited supervision, technical barriers, and digital literacy. Mixed methods integration showed that perceived technology-related barriers were associated with lower technological competence (median 1.88, IQR 1.69‐2.56 vs median 3.06, IQR 2.75‐3.59; *P*=.002), while no other significant differences emerged.

**Conclusions:**

These findings highlight that effective digital rehabilitation should be developed through iterative, user-informed HCD processes that simultaneously address cognitive, emotional, social, and technological dimensions of early neurocognitive decline. Integrated findings highlight the importance of addressing individual needs and incorporating users’ perspectives from the earliest design phases to support usable, engaging, and accessible digital rehabilitation solutions.

## Introduction

In recent years, the digital transformation of health care has created unprecedented opportunities to scale up and personalize rehabilitation interventions. Among the most impactful digital health care innovations expected to transform clinical practice, digital medicine has emerged as a key driver of change [1]. Defined as the use of digital products and services for diagnosis, prevention, monitoring, and treatment, digital medicine encompasses a broad range of technologies, including telemedicine platforms, mobile apps, sensors, and wearable devices. These solutions are already reshaping health care delivery by enabling earlier diagnosis, empowering patients in the self-management of chronic conditions, and supporting the transition of care from hospital-centered settings to patients’ homes [1].

Within this context, telemedicine has become increasingly integrated into health care systems to improve access to care, reduce nonattendance, and offer flexible alternatives to in-person consultations through the use of Information and Communication Technologies. A prominent application of telemedicine is telerehabilitation, alongside the emergence of digital therapeutics (DTx), which together represent innovative approaches for delivering evidence-based rehabilitation treatments at scale [2]. These solutions have demonstrated particular relevance in the field of cognitive rehabilitation, enabling interventions to reach broader and more diverse populations [3-8].

Telerehabilitation, which gained significant momentum during the COVID-19 pandemic [9], is defined as the remote delivery of rehabilitation services by health care professionals, often involving multidisciplinary teams and caregiver collaboration [10]. A defining feature of telerehabilitation systems is the presence of a “double-loop” communication channel between clinicians and patients, allowing for continuous and bidirectional information exchange [11]. Accumulating evidence supports both the efficiency and effectiveness of telerehabilitation in terms of adherence, clinical outcomes, and quality of life [6,7,12,13].

Complementary to telerehabilitation, DTx represents an emerging frontier in neurorehabilitation [14]. DTx are defined as high-quality health software designed to prevent, manage, or treat medical conditions by delivering interventions with demonstrable and positive therapeutic effects [15]. Despite their potential, however, the widespread adoption and sustained use of digital health solutions in neurorehabilitation remain limited. Persistent challenges related to usability, user engagement, personalization, and real-world integration continue to hinder their translation into routine clinical practice [3].

Addressing these challenges requires moving beyond a purely technology-driven approach toward the development of digital solutions that explicitly respond to the needs, preferences, capabilities, and expectations of end users, including patients, caregivers, and health care professionals [3,16,17]. This shift highlights the importance of including patient perspectives throughout the evaluation and development of digital health technologies. In this direction, the Smart&TouchID (STID) model [18] has been proposed as a structured, multidimensional, and participatory framework that embeds patients’ and stakeholders’ experiences from the earliest stages of technological development. Within the STID model, participation is conceived as an iterative process, with different forms and levels of stakeholder involvement across the various stages of technology development to support the optimization of innovative rehabilitation solutions. A common way to operationalize the co-creation principles of the STID model is through the human-centered design (HCD) methodology. The HCD approach promotes iterative development, multidisciplinary collaboration, and the active involvement of patients, caregivers, and clinicians throughout the innovation lifecycle [17]. Within this framework, data collection primarily focuses on understanding users’ needs, preferences, abilities, and contextual constraints, with the aim of informing system requirements, guiding design decisions, and identifying barriers and facilitators to adoption and sustained engagement.

Based on these premises, the present study focuses on the perspectives of individuals in the early stages of age-related neurocognitive disorders regarding the use of digital tools for cognitive rehabilitation. Neurocognitive disorders represent a major challenge for both clinical practice and public health, as they are characterized by progressive cognitive decline that interferes with daily functioning [19]. Evidence from recent meta-analyses highlights the critical role of early cognitive rehabilitation in preserving cognitive functioning and quality of life [20]. Accordingly, the preclinical and prodromal stages of dementia, namely subjective cognitive decline (SCD) and mild cognitive impairment (MCI), constitute key time windows for implementing timely, personalized, and potentially preventive rehabilitation strategies [21].

By capturing early insights into users’ needs and perceptions, this study represents a crucial preliminary needs assessment phase within a broader HCD approach. At this stage, end users were involved as key informants, contributing their lived experiences, unmet needs, perceived barriers, and expectations regarding digital cognitive rehabilitation. These insights will guide the development of clinically meaningful digital rehabilitation solutions for individuals in the prodromal and preclinical stages of dementia.

## Methods

### Overview

Guided by HCD principles, the present study involved end users as key informants, providing experiential knowledge about their cognitive difficulties, rehabilitation needs, and perceptions of digital health technologies. A mixed methods approach was considered appropriate because understanding users’ needs and perceptions required both an in-depth exploration of their subjective experiences and a quantitative characterization of their cognitive and technological profiles. Qualitative and quantitative components were given complementary roles, with neither considered predominant. To this end, a convergent mixed methods design was adopted, in which qualitative and quantitative data were collected within the same study phase and initially analyzed separately using their respective analytical approaches. The 2 data strands were subsequently jointly interpreted, focusing on areas of convergence, complementarity, and divergence between quantitative and qualitative findings [22].

To further support this integration, exploratory quantitative analyses were conducted to examine the relationships between cognitive status, self-perceived cognitive difficulties, technological familiarity and competence, and selected participants’ attitudes toward telerehabilitation derived from the qualitative coding, with the aim of translating the combined evidence into user-centered design considerations for the development of future digital rehabilitation solutions.

This study was reported in accordance with the GRAMMS (Good Reporting of a Mixed Methods Study) recommendations [23].

### Participants

Participants were recruited at the IRCCS Fondazione Don Carlo Gnocchi, Milan, using a convenience sampling strategy among individuals with SCD or MCI (N=23). Participants were recruited from a cohort of individuals who had previously participated in a telerehabilitation project (RE-ABILITY project, ID: 187469). In the RE-ABILITY project, all participants were consecutively recruited at the Memory Clinic of IRCCS Don C. Gnocchi Foundation of Milan. All individuals from this cohort who met the eligibility criteria and were available during the recruitment period were invited to participate during a clinical visit. To be enrolled, patients had to meet the following inclusion criteria: (1) diagnosis of SCD or MCI (based on the clinical diagnosis documented in participants’ outpatient neurological assessment reports), (2) age between 60 and 90 years, and (3) absence of major psychiatric disorders. Data collection and analysis were guided by the principle of thematic saturation, defined as the point at which no new themes or relevant insights emerged from the data.

### Ethical Considerations

The study was performed in accordance with the principles of the Helsinki Declaration and received prior approval from the IRCSS Fondazione Don Carlo Gnocchi ONLUS Ethics Committee (ID protocol: 2021_02_05). All participants of the study read and signed the informed consent to take part in the research. Participant data were pseudonymized and handled in accordance with applicable data protection regulations to ensure privacy and confidentiality. Participants received no compensation for their participation in the study.

### Procedure

All participants attended an individual 60-minute session involving standardized cognitive and technology-related assessments, followed by a face-to-face semistructured interview.

#### Standardized Cognitive and Technology-Related Assessments

Standardized questionnaires were administered to gather complementary quantitative data related to cognitive functioning, emotional well-being, and technology use.

Cognitive functioning was assessed through the Montreal Cognitive Assessment (MoCA) [24], a brief but comprehensive screening tool sensitive to the early stages of cognitive impairment. It evaluates multiple cognitive domains, including executive functions, attention, memory, language, visuospatial abilities, and orientation. The total score ranges from 0 to 30, with higher scores reflecting better overall cognitive performance. Raw scores were adjusted according to Italian normative data for age and education, with an adjusted cut-off score of 15.5 [24].

To measure self-perceived cognitive difficulties in everyday contexts, participants completed the Cognitive Failures Questionnaire (CFQ) [25]. This 25-item scale asks individuals to rate the frequency with which they experience lapses in memory, attention, and action, using a 5-point scale ranging from “very often” to “never.” The total score ranges from 0 to 100, with higher scores indicating greater perceived cognitive failures.

Finally, participants’ technological familiarity and perceived digital competence were assessed using an ad hoc Technological Expertise questionnaire [5]. This tool asked participants to rate, on a 5-point Likert scale, both the frequency of use and their perceived skills with 5 technologies commonly involved in home-based digital rehabilitation (smartphones, tablets, computers, gaming devices, and internet). Two separate composite scores (range 1‐5) were computed, with higher scores indicating greater digital engagement and self-confidence in using technology.

#### Semistructured Interview

The interview was designed ad hoc to elicit rich, first-person narratives concerning cognitive, emotional, and technological experiences, based on four main domains, identified through a targeted literature analysis as particularly relevant for individuals with SCD and MCI:

1. Neurocognitive abilities investigated how patients perceive their cognitive difficulties and the impact on everyday functioning. Participants were invited to describe specific everyday situations in which they experienced cognitive difficulties and to explain how they managed those episodes. They were encouraged to include contextual details (eg, location, activity) and any problem-solving strategies. Individuals with SCD and MCI may experience impairments in one or more of the 6 neurocognitive domains defined by the *DSM-5* (*Diagnostic and Statistical Manual of Mental Disorders, Fifth Edition*) [19]: learning and memory, executive function, complex attention, perceptual-motor function, language, and social cognition. These impairments present heterogeneously across patients, with differing impacts on quality of life and daily activities [26,27]. Understanding patients’ perceptions of affected domains is essential for tailoring rehabilitation interventions.
2. Network support focused on social and emotional needs, assessing changes in social roles, emotional well-being, and communication challenges. Participants discussed experiences of reduced social contact or a subjective sense of loneliness or lack of support. Social difficulties are common in MCI, including altered social roles, emotional distress, and withdrawal from social activities [28-31]. These emotional and social needs are often underdetected in clinical assessment, as patients may hesitate to disclose any difficulties due to embarrassment or uncertainty on how to express them [32-34]. Tools that capture both expressed and unspoken social and emotional needs are therefore essential for providing timely and appropriate support.
3. User experience and engagement evaluated patients’ attitudes, satisfaction, and engagement with technological solutions for rehabilitation. Participants had to share their perspective on the use of digital technology in home-based rehabilitation, focusing on their impressions, concerns/potential barriers, and perceived benefits. Promoting engagement with digital rehabilitation represents a major challenge, given its direct impact on treatment adherence and, ultimately, on the effectiveness of early interventions targeting cognitive decline [7]. Addressing disengagement requires attention to usability, acceptability, patient satisfaction, and sustainability of digital solutions [3,17,35]. Early evaluation of user experience can inform design strategies to maximize motivation and sustained participation, which are essential for successful digital therapeutic interventions.
4. Digital divide explored patients’ access to and confidence with digital tools, as well as potential barriers related to the digital divide. Disparities in digital access and competence can limit older adults’ ability to benefit from telehealth and remote rehabilitation services [36]. Beyond technological barriers, emotional and social factors, such as anxiety or lack of confidence in using new tools, may also hinder engagement. Interventions that provide training and support to improve digital literacy can help overcome these obstacles and ensure equitable participation.

These thematic areas were translated into a set of interview questions (Table 1) through an iterative, multistage development process. The initial interview draft was reviewed by a multidisciplinary panel of experts (neurologists, neuropsychologists, and rehabilitation specialists) to ensure content validity, clinical appropriateness, and linguistic accessibility. Specific attention was given to aligning the wording and structure of the questions with the characteristics of individuals with SCD and MCI, thereby minimizing cognitive load and maximizing engagement. The revised interview was then pilot-tested with a small group representative of the target population to assess clarity and accessibility from the user’s perspective. Feedback informed further refinements to question phrasing, ensuring that the final version could elicit rich and meaningful responses.

The semistructured interviews were conducted in person, in a quiet and private setting, to ensure participant comfort and facilitate open dialogue. Interviews were audio-recorded with participants’ verbal consent and subsequently transcribed verbatim by the researcher for analysis.

**Table 1.** Open-ended questions structured around four main domains.

Domain and number	Question	Collected data
Neurocognitive abilities		
1	“I’d like you to share an experience when you faced a cognitive challenge in your everyday life.”	Type of cognitive difficulty
2	“How did you deal with this difficulty?”	Problem-solving strategy
Network support		
3	“Did you tell anyone about this difficulty?”	Expressed/unexpressed need
4	“Who did you tell about your cognitive difficulty?”	Person being approached
5	“Did you feel understood or supported?”	Met/unmet need
6	“Did you consult any professionist (like a neurologist)?"	Reach out to a specialist
7	“Did the professionist suggest you a rehabilitation treatment?”	Access to the treatment
User experience and engagement		
8	“Do you know that some rehabilitative treatments can be followed remotely, for example from home, using technology?”	Knowledge about telerehabilitation
9	“In your opinion, what makes this treatment appropriate for your difficulties?”	Acceptability aspects
10	“Why do you think a telerehabilitation treatment might not be suitable?”	Nonacceptability aspects
11	“Do you think an in-person treatment would be more appropriate for your difficulties than telerehabilitation?”	Comparison between home-based rehabilitation and in-person rehabilitation
Digital divide		
12	“Do you think your tech skills might make this kind of treatment difficult?”	Technological skill impact

### Statistical Analyses

All statistical analyses were conducted using Jamovi (version 2.6.26; The Jamovi Project, 2024 [37]).

#### Quantitative Analyses

Descriptive statistics were computed to summarize demographic (age, gender, education), clinical (CFQ, MoCA), and technological characteristics. Continuous variables were reported as means and standard deviations (SDs), while categorical variables were expressed as frequencies and percentages. The normality of continuous variables was assessed using skewness and kurtosis values.

#### Qualitative Content Analysis

The verbatim transcription of the open-ended answers was qualitatively analyzed using a directed content analysis approach. Two psychologists with expertise in neurodegenerative conditions and cognitive rehabilitation independently reviewed the full transcripts and identified coding units corresponding to the four thematic areas identified a priori: (1) digital divide, (2) neurocognitive abilities, (3) network support, and (4) user experience and engagement. Following initial data familiarization, the 2 raters performed independent coding in a double-blind manner. Interrater agreement varied across coding categories, with Cohen κ values ranging from 0.40 to 0.95, indicating fair-to-almost-perfect agreement [38].

Disagreements were discussed and resolved by consensus. This approach was designed to enhance analytic rigor and maintain alignment with the predefined conceptual framework. Credibility was supported by independent coding followed by consensus discussion; dependability by the systematic use of the same interview guide and analytical framework across participants; and confirmability by independent coding prior to consensus and by grounding the analysis in participants’ verbatim responses.

#### Mixed Methods Integration

Exploratory analyses were conducted to examine the relationships between cognitive status (MoCA), self-perceived cognitive difficulties (CFQ), technological familiarity and competence, and participants’ attitudes toward telerehabilitation. MoCA, CFQ, technological familiarity, and technological competence were compared according to 3 qualitatively derived binary categories: motivation and engagement, based on whether a positive perception of independently doing something for one’s own well-being emerged from participants’ narratives (“positive perception emerged/not emerged”); perceived lack of in-person interaction and direct supervision (“concern emerged/not emerged”); and perceived technology-related barriers to adherence (“barrier perceived/not perceived”), using Mann-Whitney *U* tests. To account for multiple comparisons across the exploratory analyses, a Bonferroni-adjusted significance threshold of *P*<.0042 was applied.

## Results

### Overview

Thematic saturation was achieved after 23 interviews, as subsequent interviews did not yield new themes and provided only confirmatory evidence for the themes already identified. The demographic, clinical, and technological expertise characteristics of the included sample (N=23) have been described by reporting means, SDs, and frequencies (Table 2).

Interrater agreement across coding categories was fair to almost perfect, with Cohen κ values ranging from 0.40 to 0.95 [38]. The findings from the semistructured interview are reported in Table 3 and in the following sections, structured by each of the 4 thematic domains explored.

**Table 2.** Quantitative data: demographic and clinical characteristics of the sample.

Characteristics	Values
Age (y), mean (SD; range)	74.3 (7.35; 54‐85)
Education (y), mean (SD; range)	11.7 (4.37; 5‐18)
Sex, n	
Male	7
Female	16
MoCA^a^ (adjusted score; cut-off=15.5), mean (SD; range)	24.9 (3.23; 19.9‐30)
CFQ^b^, mean (SD; range)	22.4 (9.48; 8‐44)
Technological expertise, mean (SD; range)	
Familiarity	2.97 (0.76; 1.25‐4.25) (weekly use)
Competence	2.85 (0.83; 1.13‐4.50) (moderate-to-low)

a MoCA: Montreal Cognitive Assessment Test.

b CFQ: Cognitive Failures Questionnaire.

**Table 3.** Qualitative data: findings from the semistructured interview.

Domain, collected data, and results	Interviews, n/N (%)
Neurocognitive abilities	
Type of cognitive difficulty	
Memory issues	12/23 (52.2)
Sustained attention	6/23 (26.1)
Language difficulties	4/23 (17.4)
Problem-solving strategy	
Minimizing or denying difficulties	5/23 (21.7)
Compensatory strategies, such as writing reminders or avoiding complex tasks	8/23 (34.8)
No strategy	3/23 (13)
Support request	7/23 (30.4)
Network support	
Expressed/unexpressed need	
Sharing difficulties with family or friends	17/23 (73.9)
Person being approached	
Wife/husband	8/17 (47.1)
Son/daughter	6/17 (35.3)
Friend	1/17 (5.9)
Met/unmet need	
Concerns acknowledged	14/17 (82.4)
Emotional unsupported	3/17 (17.6)
Reach out to a specialist	
Yes	15/23 (65.2)
Access to the treatment	
Concerns dismissed by health care professionals	15/23 (65.2)
Delayed interventions	14/15 (93.3)
User experience and engagement	
Knowledge about telerehabilitation	
Digital solution potential recognized	6/23 (26.1)
Acceptability aspects	
Telerehabilitation as convenient, accessible, and sustainable solution	21/23 (91.3)
Perceived usability	16/23 (69.6)
Nonacceptability aspects	
Technical issues	4/23 (17.4)
Comparison between home-based rehabilitation and in-person rehabilitation	
Missing in-person interaction and supervision	10/23 (43.5)
Digital divide	
Technological skill impact	
No prior knowledge	17/23 (73.9)
Tech could affect adherence	7/23 (30.4)

### Neurocognitive Abilities

Distinct patterns of neurocognitive difficulties emerged from the interviews, with 12 out of 23 (52.2%) participants reporting memory-related issues consistent with the Learning and Memory domain described in the DSM-5. The most commonly reported difficulties included forgetting appointments and confusion about the temporal sequence of events. One participant explained: “What happened to me today—and on many other occasions—is that I couldn’t remember what day or time an appointment was, so I had to check again. It stressed me out, and I get upset when I miss appointments.” Another recalled a similar situation: “Once, I showed up a week early for a medical appointment, taking someone else’s spot, because I had forgotten it was scheduled for the following week.” A third participant shared: “I once went to my music lesson, rang the doorbell, and nobody answered. I sat there worried until I suddenly remembered my teacher had said we weren’t meeting that day. I went home trying to tell myself it wasn’t a big deal…” These accounts reflect how memory difficulties, particularly those related to managing time and appointments, can lead to stress, confusion, and a sense of disorientation in daily life.

Six out of 23 (26.1%) participants also described difficulties with sustained attention, managing multiple tasks, and switching between activities—challenges aligned with *Executive Function* and *Complex Attention* impairments. These issues interfered with daily routines and often left participants feeling overwhelmed. One participant reflected: “Nothing specific comes to mind now… maybe when I’m cooking, I don’t always remember the ingredients, so I have to check the recipe, which I used to do automatically.” Another noted: “At work, if someone interrupts me, I find it hard to regain concentration, but it’s nothing too serious.” A third added*:* “I took on collecting money from six or seven people, but in the end, I got totally confused with the numbers—who owed what and how much I had to give back.” Such experiences often left participants feeling overwhelmed, even when the tasks involved were relatively simple or familiar.

In addition, 4 out of 23 (17.4%) participants described difficulties specifically related to language, particularly word-finding problems. These episodes were often experienced as sudden and frustrating interruptions in communication, especially during everyday conversations. One participant described her experience with a family member, highlighting how word-finding difficulties can trigger negative reactions from others: “Sometimes I can’t find the right word, especially when talking to my granddaughter or daughter. Then she says, ‘Mom, are you with us or not?’”

This type of response left participants feeling embarrassed and uncomfortable and, in some cases, raised concerns about the progression of their difficulties. In some cases, these lapses occurred in public or social contexts, heightening feelings of vulnerability and embarrassment. As one participant recalled:

*This morning I bumped into a neighbor and told her, “I’m heading to…” — and I completely blanked on the name of the street. I felt so embarrassed and it really bothered me. I don’t know what to do. Honestly, I can tell I’m getting worse at this, and that scares me.*

These narratives illustrate how even mild linguistic difficulties can undermine self-confidence, trigger fear of decline, and negatively influence social interactions, highlighting the importance of considering emotional responses and perceived stigma when designing supportive interventions for early cognitive changes.

Finally, 5 out of 23 participants (21.7%) appeared to minimize or deny the presence of cognitive difficulties. In these cases, cognitive lapses were often attributed to “normal aging” rather than to any pathological process. However, while some explicitly stated they did not experience any issues, their narratives revealed the frequent use of compensatory strategies, such as writing reminders or avoiding complex tasks, suggesting an implicit awareness of subtle changes. This form of minimization may reflect coping mechanisms such as denial or normalization, which can serve to protect one’s self-image and reduce anxiety in the face of perceived decline. However, it also poses challenges for early detection and intervention, as individuals who downplay their symptoms may delay seeking help or fail to engage fully with available rehabilitation programs.

### Network Support

Participants described reduced social interactions due to situational factors such as having a limited social network and low participation in social groups. One participant shared: “I miss companionship and feel the need for friendships, but I’m a bit embarrassed to ask. Even with my siblings, I know they’re busy, so I tend to withdraw.”

Most participants (17/23, 73.9%) reported sharing their difficulties with family or friends, and in 82.6% of these cases (14/17), they felt their concerns were acknowledged. However, 6 out of 23 (26.1%) participants reported feeling emotionally unsupported and struggled to express their needs. These feelings were often intensified by a sense of being misunderstood or overlooked. For instance, one participant said: *“*My wife gets angry and scolds me because, due to my forgetfulness, she always has to do everything herself.” Another reflected: “Sometimes, even the people closest to me can’t truly understand what I’m dealing with—it feels like I’m going through it alone.”

These communication challenges can significantly hinder the timely recognition of emerging needs and the implementation of appropriate interventions. Of particular concern is that 65.2% (15/23) of the participants who raised cognitive concerns with health care professionals felt their experiences were dismissed or inadequately addressed, often leading to frustration and a sense of helplessness. As one participant explained: “I keep trying to tell them, but...they don’t seem to understand what I need. It’s like they’re not even listening.” Another noted: “I told the doctor what’s been going on, but he just said we’ll check again in a year. It feels like he doesn’t think it’s urgent.”

Even in cases where health care providers acknowledged patients’ concerns, immediate rehabilitation or support options were rarely offered. Strikingly, 14 out of 15 (93.3%) participants of those who received some form of acknowledgment still did not receive timely interventions, despite expressing clear emotional distress or a decline in daily functioning.

### User Experience and Engagement

Overall, perceptions of telerehabilitation were largely positive. Most participants reported feeling satisfied and supported throughout the process, despite some initial skepticism or discomfort. One participant admitted: “At first, I missed having someone physically present,” while another questioned: “What’s the point if it’s just me and the computer anyway?”

Six out of 23 (26.1%) participants explicitly acknowledged the potential of digital tools to address cognitive challenges. Convenience and practicality emerged as central themes. In fact, 21 out of 23 (91.3%) participants identified telerehabilitation as a convenient, accessible, and sustainable alternative, particularly for those with mobility issues or limited access to clinics. As one participant put it: “The benefit of home-based rehabilitation is that you don’t have to go anywhere; you can do it from your own home.” Others emphasized the comfort of the home setting:

*Doing the program at home is reassuring because you’re in your own environment—it’s something new, but in your own space.*

*Home is where I always feel most comfortable.*

*Going there three times a week? No, thanks—I can make time for 40 minutes at home.*

*Attending in person would triple the amount of time needed.*

*You can do it in shorts without any shame since you’re at home—just lock yourself in a room, and it’s no big deal.*

Ease of use was another key advantage. Although many participants initially felt uncertain, 69.6% (16/23) found the telerehabilitation system intuitive and easy to navigate. For 9 out of 23 (39.1%) participants, the sense of doing something independently for their own well-being played a crucial role in maintaining motivation and engagement. One participant shared: “I felt motivated to do something and relieved from my laziness.” Another added: “This time, I said, ‘Okay, I’m doing something just for myself,’ and it feels good.” Flexibility in scheduling and the ability to fit sessions into daily routines were also highly appreciated, helping to reduce barriers and improve adherence. As one participant noted: “If I can’t do it now, I’ll do it later.” Others remarked: “A benefit is the flexible timing—you can do it at lunch, before work, or after work.” *“*You can do it wherever you want, including the beach or mountains if you have the opportunity.”

Despite the overwhelmingly positive feedback, 4 out of 23 (17.4%) of the participants reported technical issues, such as unstable internet connections or uncertainty around who should bear the cost of necessary equipment, as limiting factors: *“*Not everyone has internet, and even when they do, it’s not always stable.”

Moreover, 10 out of 23 (43.5%) participants expressed unease about the lack of in-person interaction and the absence of direct supervision. For some, these aspects reduced both motivation and the perceived effectiveness of the intervention. Participants observed:

*The drawback is that without oversight, if I’m not motivated, I tend to skip it or go easy.*

*Without someone physically present, I’m less likely to follow through.*

*No one checks if you’re actually doing it…that makes a difference.*

### Digital Divide

Seventeen out of 23 (73.9%) participants had no prior knowledge of telerehabilitation before their involvement in the program, and 30.4% (7/23) indicated that technology use could potentially impact their ability to adhere to a home-based rehabilitation program, highlighting barriers to treatment engagement.

Participants’ narratives reflected a wide range of experiences, from dependence on family members to feelings of inadequacy or discomfort:

*I don’t know how to use the tablet. Last time they gave it to me, my daughter helped, but she couldn’t come twice a week. I just don’t get along with technology. I would need to learn, maybe with the help of my 10-year-old granddaughter.*

Others emphasized the generational gap and lack of technological education during their youth: “I was born 83 years ago, so back then we were taught very different things.” One participant humorously described their tech skills as: “My tech level is…orangutan-oriented. Like, ‘Oh God, what do I do now?’”

Given these barriers, it is essential to implement structured support strategies, such as dedicated training sessions at the clinical center, involving caregivers, when possible, to help patients and their families become familiar with the technology and improve usability, confidence, and adherence.

### Mixed Methods Integration

To integrate the qualitative and quantitative findings, we examined whether participants’ perceptions of telerehabilitation were associated with their cognitive and technological profiles (MoCA, CFQ, and Technological Expertise Questionnaire [TEQ]; Tables 4-6).

No significant differences in cognitive status, self-perceived cognitive difficulties, technological familiarity, or technological competence were observed between participants who reported a positive perception of telerehabilitation in relation to motivation and engagement and those for whom this positive perception did not emerge (MoCA: rrb=0.135; *P*=.61; CFQ: rrb=0.143; *P*=.59; technological familiarity: rrb=0.214; *P*=.41; technological competence: rrb=0.143; *P*=.59). A similar pattern was observed for concerns regarding the lack of in-person interaction and direct supervision, with no significant between-group differences in MoCA (rrb=0.008; *P*>.99), CFQ (rrb=−0.185; *P*=.47), technological familiarity (rrb=0.031; *P*=.93), or technological competence (rrb=0.015; *P*=.98).

In contrast, participants who perceived technology use and/or technological skills as a potential barrier to adherence showed significantly lower technological competence than those who did not perceive such barriers (median2.88, IQR 2.50-3.63 vs median 3.06, IQR 2.78-3.44, rrb=−0.848; *P*=.002), with this difference remaining significant after correction for multiple comparisons. Technological familiarity showed a similar pattern (rrb=−0.491; *P*=.07), although the difference did not reach statistical significance. No other significant between-group differences were observed (Tables 4-6).

**Table 4.** Mixed methods integration: exploratory comparisons of cognitive and technological characteristics according to participants’ qualitatively derived perceptions of telerehabilitation^a^.

Qualitatively derived category/variable	Positive perception not emerged (n=14), median (IQR)	Positive perception emerged (n=9), median (IQR)	Mann-Whitney *U* test	*P* value (unadjusted)	*P* value (adjusted)	r_rb
Motivation and engagement facilitators						
MoCA^b^	24.7 (22.1‐26.9)	25.0 (23.8‐27.6)	54.5	.61	ns^c^	0.135
CFQ^d^	21.5 (13.3‐28.3)	25.0 (23.0‐26.0)	54.0	.59	ns	0.143
Tech_familiarity	2.94 (2.31‐3.47)	3.13 (2.88‐3.63)	49.5	.41	ns	0.214
Tech_competence	2.81 (2.41‐3.13)	2.75 (2.63‐3.88)	54.0	.59	ns	0.143

a Between-group comparisons were performed using Mann-Whitney *U* tests. r_rb=rank-biserial correlation effect size. A Bonferroni-adjusted significance threshold of *P*<.0042 was applied to account for 12 exploratory comparisons.

b MoCA: Montreal Cognitive Assessment.

c ns: not specified.

d CFQ: Cognitive Failures Questionnaire.

**Table 5.** Mixed methods integration: exploratory comparisons of cognitive and technological characteristics according to participants’ qualitatively derived perceptions of the lack of in-person interaction and supervision in telerehabilitation^a^.

Qualitatively derived category/variable	Concern not emerged (n=13), median (IQR)	Concern emerged (n=10), median (IQR)	Mann-Whitney *U* test	*P* value (unadjusted)	*P* value (adjusted)	r_rb
Perceived lack of in-person interaction/supervision						
MoCA^b^	24.9 (23.8-26.9)	25.0 (21.4-27.4)	64.5	>.99	ns^c^	0.008
CFQ^d^	25.0 (14.0-29.0)	23.0 (16.5-24.8)	53.0	.47	ns	–0.185
Tech_familiarity	2.88 (2.50-3.63)	3.06 (2.78-3.44)	63.0	.93	ns	0.031
Tech_competence	2.75 (2.50-3.25)	2.75 (2.56-3.09)	64.0	.98	ns	0.015

a Between-group comparisons were performed using Mann-Whitney *U* tests. r_rb=rank-biserial correlation effect size. A Bonferroni-adjusted significance threshold of *P*<.0042 was applied to account for 12 exploratory comparisons.

b MoCA: Montreal Cognitive Assessment.

c ns: not specified.

d CFQ: Cognitive Failures Questionnaire.

**Table 6.** ^a^Mixed methods integration: exploratory comparisons of cognitive and technological characteristics according to participants’ qualitatively derived perceptions of technology-related barriers to telerehabilitation adherence.

Qualitatively derived category/variable	Barrier not perceived (n=16), median (IQR)	Barrier perceived (n=7), median (IQR)	Mann-Whitney *U* test	*P* value (unadjusted)	*P* value (adjusted)	r_rb
Perceived technology-related issues to adherence						
MoCA^b^	25.1 (23.4‐27.0)	24.5 (21.8-26.1)	47.5	.59	ns^c^	−0.152
CFQ^d^	24.5 (14.5-‐26.0)	22.0 (17.5-24.0)	50.0	.71	ns	−0.107
Tech_familiarity	3.13 (2.84-3.63)	2.38 (1.75-3.06)	28.5	.07	ns	−0.491
Tech_competence	3.06 (2.75-3.59)	1.88 (1.69-2.56)	8.5	.002	.02	−0.848

a Between-group comparisons were performed using Mann-Whitney *U* tests. r_rb=rank-biserial correlation effect size. A Bonferroni-adjusted significance threshold of *P*<.0042 was applied to account for 12 exploratory comparisons.

b MoCA: Montreal Cognitive Assessment.

c ns: not specified.

d CFQ: Cognitive Failures Questionnaire.

## Discussion

This study aimed to explore the needs, experiences, and expectations of individuals with MCI and SCD regarding digital rehabilitation tools by adopting an HCD perspective. The results shed light on 4 key domains—neurocognitive functioning, socialization, user experience/engagement, and digital divide—offering a multidimensional view of early-stage cognitive decline and its rehabilitation needs.

### Cognitive Challenges in Everyday Functioning

Memory deficits, executive and attentional difficulties, and occasional language lapses emerged as the most commonly reported cognitive complaints, aligning with the DSM-5-defined domains typically affected in individuals with MCI [19]. Notably, participants described a variety of internal (eg, repetition, mental cues) and external (eg, notes, calendars) compensatory strategies, demonstrating a degree of metacognitive awareness and adaptability and a desire to maintain autonomy. To effectively personalize interventions, it is essential to consider how these difficulties impact patients’ daily functioning and overall quality of life [39]. This highlights the need for tools capable of promptly identifying such challenges, to enable early and targeted responses. Specifically, our findings underscore the value of designing rehabilitation tools that leverage patients’ existing coping mechanisms, fostering autonomy, and reinforcing self-efficacy. Furthermore, they highlight the importance of embedding interventions within ecological, real-world contexts to enhance both relevance and transferability to everyday life [40]. These findings are consistent with previous studies showing that individuals with SCD and MCI prioritize maintaining independence in everyday activities and value interventions that address the practical consequences of cognitive decline in daily life [41]. Incorporating these patient priorities into the early stages of intervention development may help ensure that future digital rehabilitation solutions address outcomes that are meaningful and relevant to end users.

### Social and Emotional Dimensions: The Hidden Need

Social withdrawal, unmet emotional needs, and difficulty expressing own vulnerability were recurrent themes, echoing the literature on the “invisible burden” of early-stage cognitive disorders [29,32]. This finding is consistent with previous literature showing that, beyond cognitive functioning, individuals living with cognitive impairment value outcomes related to quality of life, autonomy, meaningful activities, social relationships, and emotional well-being [42]. Despite the centrality of social functioning to quality of life, these aspects remain poorly captured in traditional clinical assessments. Our findings suggest that digital interventions should incorporate features aimed at fostering social connectedness, addressing both cognitive and emotional rehabilitation needs.

### Unmet Needs and Delayed Access to Rehabilitation

Consistent with previous findings [43], our study confirms a persistent gap in the early-stage clinical management of cognitive complaints. Although most participants had shared their cognitive concerns with health care professionals, the majority reported receiving limited feedback or recommendations, with rehabilitative options rarely discussed. This aligns with evidence indicating that non-pharmacological interventions are under-prescribed despite their proven efficacy in the prodromal phases of dementia [44,45]. Notably, participants’ voluntary involvement in a research-based rehabilitation program may reflect a proactive effort to compensate for this clinical void. These findings underscore the need to systematically integrate early rehabilitation into clinical practice, supported by innovative digital health technologies.

### Positive Perception of Telerehabilitation: Conditions for Engagement

Most participants described the telerehabilitation experience as positive, highlighting convenience, personalization, and the sense of “doing something for themselves” as key facilitators of adherence. These findings align with literature identifying user satisfaction, personal relevance, and perceived benefit as key engagement drivers [46]. However, some users expressed concern about the lack of direct supervision. This highlights the value of embedding continuous support mechanisms—such as remote coaching or automated feedback loops—within the intervention, reinforcing motivation and treatment adherence through the “double-loop” communication model [11].

### The Digital Divide: A Barrier That Can Be Bridged

Although many participants initially reported technological unfamiliarity and insecurity, a clear willingness to engage with digital tools also emerged. This finding calls into question the common assumption that older adults are inherently resistant to technology and reinforces recent evidence suggesting that perceived digital barriers can be effectively reduced by adopting a user-centered design approach from the earliest stages of implementation [3,47,48], as well as through the active involvement of caregivers [3,48]. Notably, users did not reject technology per se but expressed some concerns around autonomy, usability, and logistical issues such as internet access or device costs. These insights suggest that the successful implementation of telerehabilitation depends less on preexisting digital literacy and more on the usability and acceptability of the digital intervention. These implications should nevertheless be interpreted in light of participants’ previous involvement in a telerehabilitation project, which may have influenced their familiarity with and attitudes toward digital rehabilitation.

### Mixed Methods Integration

The integrated quantitative and qualitative findings suggest that participants’ attitudes toward telerehabilitation cannot be explained solely by their cognitive or technological profiles. In particular, perceptions related to motivation, engagement, and the need for human interaction appear to reflect broader dimensions of the user experience, rather than being simply determined by individual cognitive abilities or technological skills. In this respect, the qualitative findings complemented the quantitative results by capturing dimensions of the user experience that were not clearly associated with the cognitive and technological characteristics examined. This interpretation is consistent with our previous findings, showing that intention to use telerehabilitation was associated with perceived usefulness and perceived ease of use, while technological expertise and education were not significantly associated with these core technology-acceptance beliefs in the overall sample [3].

A convergent pattern emerged when considering potential technology-related critical issues affecting adherence. Participants who perceived technology as a potential obstacle to adherence also showed lower technological competence, indicating convergence between their subjective perceptions and measured technological skills. This finding suggests that technological competence may not necessarily shape how users perceive the value of telerehabilitation but may become relevant when technology itself represents a barrier to its continued use. No clear patterns of divergence between the qualitative and quantitative findings emerged from the integrated analysis.

### Implications for Design and Practice

This distinction has practical implications for the development of digital rehabilitation solutions, as different dimensions of the user experience may require different design and support strategies. Accordingly, the findings of this study translate into several practical recommendations for designing and implementing digital rehabilitation tools that are clinically effective, emotionally resonant, and technologically accessible.

First, personalization should be a guiding principle. Interventions should not only be tailored to patients’ actual performance but also to their self-perceived difficulties and confidence in their abilities, to bridge the gap created by the digital divide [49]. Moreover, as emerged from the interviews, personalization should reflect not only individual cognitive profiles but also the social and emotional needs of each user. This involves adapting both the content and structure of the rehabilitation program to ensure it is both meaningful and manageable in everyday life.

Equally important is the provision of structured support and training. Offering guided onboarding sessions and involving caregivers can play a crucial role in reducing digital anxiety and building user confidence, particularly at the beginning of the rehabilitation program.

To sustain motivation and address users’ need for human interaction, continuous feedback mechanisms should be embedded, ensuring regular, bidirectional communication between users and healthcare professionals, according to the “double-loop” communication model [11]. Such support may be especially relevant for users with lower technological competence, for whom technology itself may represent a barrier to sustained engagement.

Finally, digital interventions should be flexible enough to allow users a certain degree of autonomy, enabling them to decide when and how to engage with the program while remaining within a safe and supportive framework. Integrating patients’ voices and lived experiences into the design process, as promoted by the HCD approach, not only improves usability but also ensures that the solutions are therapeutically meaningful, emotionally resonant, and more likely to be accepted and sustained in real-world contexts [17]. Moreover, these findings reflect the multidimensional and participatory evaluation logic promoted by the STID model, highlighting how cognitive, emotional, social, and technological dimensions jointly shape the acceptability and sustainability of digital rehabilitation solutions in early-stage cognitive decline. Importantly, these implications are particularly relevant in the early stages of digital therapeutic development, when incorporating user feedback can prevent downstream usability and engagement issues.

These findings should be interpreted as the first step of an iterative HCD pathway. By incorporating end users’ perspectives during the needs assessment phase, this study provides the empirical foundation for defining user requirements that will inform the subsequent development and evaluation of future digital rehabilitation solutions.

This study has several limitations. The sample size was relatively small and may not capture the full heterogeneity of experiences in the broader SCD/MCI population. The small sample size also limited the statistical power of the exploratory mixed methods analyses, particularly given the unequal size of the groups derived from the qualitative categories. Therefore, exploratory results should be confirmed in larger samples. Participants had prior exposure to digital tools through research involvement, potentially biasing attitudes toward telerehabilitation. Individuals with SCD or MCI without previous experience with digital rehabilitation may face difficulties related to digital literacy [50], potentially resulting in less favorable perceptions of telerehabilitation than those observed in our sample. Future studies should therefore include more diverse populations, particularly individuals with limited prior exposure to digital rehabilitation and varying levels of technological familiarity and competence, and examine longitudinal adherence and outcomes during real-world implementation. Additionally, because the primary aim of this exploratory needs assessment was to generate initial user insights to inform the early HCD phase, participants were not purposively selected based on socioeconomic characteristics. Since these factors are known to influence access to and adoption of digital health technologies [51], future HCD studies should consider deliberately including participants with diverse socioeconomic characteristics to better capture the needs of populations at higher risk of digital exclusion. Future research should examine the transferability of these findings across different settings, as this single-center Italian study may reflect context-specific health care, cultural, and technological factors. Multicenter and cross-cultural studies involving populations with varying levels of digital literacy are needed to assess their applicability across different health care systems and sociocultural contexts. Finally, integrating clinician perspectives in the next design phases will be essential to ensure clinical feasibility and workflow integration.

### Conclusions

This study provides an in-depth exploration of the needs, expectations, and lived experiences of individuals with SCD and MCI in relation to digital cognitive rehabilitation. The integration of qualitative and quantitative findings further suggests that telerehabilitation acceptance is shaped by factors extending beyond users’ cognitive and technological profiles, while technological competence may become relevant when technology itself represents a potential barrier to adherence.

Consistent with HCD principles and the participatory, multidimensional approach of the co-creation STID model, these findings underscore the need for digital rehabilitation solutions grounded in iterative, user-informed processes that address the cognitive, emotional, social, and technological dimensions of early cognitive decline. Importantly, moving beyond preconceived assumptions about the suitability of digital rehabilitation for individuals with cognitive or technological difficulties represents a critical step toward more inclusive and equitable rehabilitation models. Designing digital solutions around users’ actual needs and capabilities, rather than presumed limitations, may ultimately contribute to expanding access to rehabilitation and advancing the goal of rehabilitation for all.

## Abbreviations

CFQ: Cognitive Failures Questionnaire
*DSM-5*: *Diagnostic and Statistical Manual of Mental Disorders, Fifth Edition*
DTx: digital therapeutics
GRAMMS: Good Reporting of a Mixed Methods Study
HCD: human-centered design
MCI: mild cognitive impairment
MoCA: Montreal Cognitive Assessment
SCD: subjective cognitive decline
SD: standard deviation
STID: Smart&Touch-ID
TEQ: Technological Expertise Questionnaire
